# Predictors of Postal or Online Response Mode and Associations With Patient Experience and Satisfaction in the English Cancer Patient Experience Survey

**DOI:** 10.2196/11855

**Published:** 2019-05-02

**Authors:** Tra My Pham, Gary A Abel, Mayam Gomez-Cano, Georgios Lyratzopoulos

**Affiliations:** 1 Epidemiology of Cancer Healthcare and Outcomes Group Department of Behavioural Science and Health University College London London United Kingdom; 2 University of Exeter Medical School (Primary Care) University of Exeter Exeter United Kingdom

**Keywords:** cancer, patient survey, satisfaction, experience, online response, Web, internet

## Abstract

**Background:**

Patient experience surveys are important tools for improving the quality of cancer services, but the representativeness of responders is a concern. Increasingly, patient surveys that traditionally used postal questionnaires are incorporating an online response option. However, the characteristics and experience ratings of online responders are poorly understood.

**Objective:**

We sought to examine predictors of postal or online response mode, and associations with patient experience in the (English) Cancer Patient Experience Survey.

**Methods:**

We analyzed data from 71,186 patients with cancer recently treated in National Health Service hospitals who responded to the Cancer Patient Experience Survey 2015. Using logistic regression, we explored patient characteristics associated with greater probability of online response and whether, after adjustment for patient characteristics, the online response was associated with a more or less critical evaluation of cancer care compared to the postal response.

**Results:**

Of the 63,134 patients included in the analysis, 4635 (7.34%) responded online. In an adjusted analysis, male (women vs men: odds ratio [OR] 0.50, 95% confidence interval [CI] 0.46-0.54), younger (<55 vs 65-74 years: OR 3.49, 95% CI 3.21-3.80), least deprived (most vs least deprived quintile: OR 0.57, 95% CI 0.51-0.64), and nonwhite (nonwhite vs white ethnic group: OR 1.37, 95% CI 1.24-1.51) patients were more likely to respond online. Compared to postal responders, after adjustment for patient characteristics, online responders had a higher likelihood of reporting an overall satisfied experience of care (OR 1.24, 95% CI 1.16-1.32). For 34 of 49 other items, online responders more frequently reported a less than positive experience of care (8 reached statistical significance), and the associations were positive for the remaining 15 of 49 items (2 reached statistical significance).

**Conclusions:**

In the context of a national survey of patients with cancer, online and postal responders tend to differ in their characteristics and rating of satisfaction. Associations between online response and reported experience were generally small and mostly nonsignificant, but with a tendency toward less than positive ratings, although not consistently. Whether the observed associations between response mode and reported experience were causal needs to be examined using experimental survey designs.

## Introduction

Patient experience is an important aspect of quality of care [1]. In England, the National Health Service (NHS) has embarked on major policy initiatives regarding the measurement of patient experience through nationwide surveys since 2009 [2,3]. Relatedly, the National Cancer Strategy indicates that patient experience should be considered as “being on a par with clinical effectiveness and safety” [4].

The English Cancer Patient Experience Survey (CPES), a major nationwide survey of patients recently treated for cancer, was first undertaken in 2010. Internationally, it is the largest annual nationwide survey of patients with cancer [5]. Survey items assess key domains of patient experience [6-8], including the interpersonal skills of care providers, the provision of information about cancer diagnosis and its treatment, and the experience of access and timeliness of care, care coordination, and satisfaction with care. Its findings are reported publicly, both at the national level and for each hospital and health care commissioning organization. The survey has consistently had a high response rate across different waves (approximately 65%-67%) [9].

The CPES was chiefly a postal survey until 2015 when an online response option was introduced. In this new context, it is important to understand which groups of patients tend to use the online response mode, and whether patients using different response modes rate their care experience differently. Such understanding can help to establish whether comparisons between patient groups or between hospitals and over time might be impacted by the introduction of the online response option and variation in its use [10].

With these prior considerations, we aimed to examine the sociodemographic and cancer site predictors of the postal or online response mode in CPES 2015 and, subsequently, to examine the associations between response mode and key aspects of the cancer patient experience.

## Methods

### Data Source

We analyzed anonymous data from responders to CPES 2015 [11]. The survey was commissioned by NHS England and carried out by Quality Health, a specialist survey provider. The sampling frame consisted of patients aged 16 years and older who were treated for cancer in English NHS hospitals from April to June 2015. After relevant vital status checks, patients were mailed the questionnaire (with two reminders for nonresponders). Patients could complete and return the survey questionnaire by post or complete it online. The questionnaire could also be completed by phone via a freephone helpline, which also provided a translation and interpreting facility for patients whose first language was not English. Of the 108,269 initially sampled eligible patients, 71,186 completed the questionnaire (response rate=65.75%).

### Variables

Information was available on responders’ age group (<55 years, 55-64 years, 65-74 years, ≥75 years), sex, self-reported ethnic group (white, nonwhite), and deprivation status (based on quintiles of the Index of Multiple Deprivation [IMD] scores of the lower super output area of patients’ residence) [12]. Patients’ cancer diagnoses were categorized into the 11 major cancer sites (endometrial, melanoma, leukemia, rectal, lung, non-Hodgkin lymphoma, bladder, colon, multiple myeloma, prostate, breast) and an “other” group using the *International Classification of Diseases, Tenth Revision*, diagnosis code (based on hospital records) [13]. To ensure conformity with a strict anonymization standard (ie, regarding the minimum count of patients in a given stratum), ethnicity information in our analysis sample was suppressed by the data provider for 3064 (4.30%) patients; these were patients with melanoma, renal, and thyroid cancers (Multimedia Appendix 1). Information on the survey response mode was also available and categorized into three groups (postal, online, and other).

The survey consisted of 49 evaluative questions reflecting different aspects of the cancer care experience, with a question on overall satisfaction with care (#59: “Overall, how would you rate your care?”) (M Gomez-Cano et al, unpublished data, 2019). Of the 49 evaluative questions, 7 had binary response options and 42 used a Likert-response format. We used binary forms of the latter items (“positive” and “less than positive” experience categories), consistent with public reporting conventions of the survey [14]. Overall satisfaction with care was rated using scores 0 (very poor) to 10 (very good); answers to this question were dichotomized into two categories of “satisfied” (scores 9 or 10) or “less than satisfied” (scores 0 to 8). We were a priori interested in the question regarding overall satisfaction with cancer care (#59) separately to the other (experience) items, considering satisfaction as an outcome of care experience (M Gomez-Cano et al, unpublished data, 2019).

We excluded individuals who responded to the questionnaire with modes other than postal or online, had missing or suppressed ethnic group, or had missing deprivation information (ie, information on sex and age group was complete). Data from 63,134 responders were used for all analyses, representing 88.69% of the total responders’ sample (Multimedia Appendix 2). For associations between response mode and reported experience, responders with missing or noninformative answers (eg, “don’t know/can’t say”) to the survey questions were further excluded, resulting in variation in sample size across the different questions.

### Statistical Analyses

To examine predictors of online response, crude proportions of responders using the postal and online response modes were calculated by sociodemographic characteristic and cancer site variables. Univariable and multivariable logistic regression models were used to obtain (1) the unadjusted odds ratios (ORs) of online response, and (2) the ORs of online response adjusted for all patient characteristic (age group, sex, ethnic group, deprivation status) and cancer site variables considered. From the latter model, covariate-adjusted proportions of online responders were predicted and compared with the corresponding crude proportions.

Similarly, to examine the associations between postal or online response mode and reported experience, for each of the 50 survey questions, univariable and multivariable logistic regression models were used to obtain (1) the unadjusted ORs of reporting a satisfied/positive experience for response mode, and (2) the OR of reporting a satisfied/positive experience for response mode, adjusted for all patient characteristic and cancer site variables considered.

### Supplementary Analyses

In supplementary analyses examining the association between response mode and overall satisfaction with care (#59), the sensitivity of the main analysis findings to another cut-off choice was explored, using scores 8 to 10 for a satisfied experience instead of scores 9 or 10 as in the main analysis. For this item, we also examined pairwise interactions between response mode and each sociodemographic variable in the multivariable logistic regression model. All analyses were conducted using Stata version 15.1 (StataCorp LP, College Station, TX, USA).

## Results

### Predictors of Online Response

Of the 63,134 patients included in this analysis, 58,499 (92.66%) completed the survey by post and 4635 (7.34%) online. In univariable analyses, online response mode was less likely among women (OR 0.68, 95% confidence interval [CI] 0.64-0.72 for women versus men) (Table 1). Increasing age was associated with lower likelihood of online response (OR 0.40, 95% CI 0.36-0.44 for ≥75 versus 65-74 years). Increasing level of deprivation was similarly associated with lower likelihood of online response (OR 0.70, 95% CI 0.63-0.78 for IMD quintile 5 versus quintile 1). Nonwhite patients were more likely to respond online compared to white patients (OR 1.62, 95% CI 1.47-1.78). There was also evidence for variation in the odds of online response between patients across different cancer sites (joint *P* value <.001), with leukemia associated with the greatest odds and lung cancer with the lowest odds of online response compared with rectal cancer.

**Figure 1:**
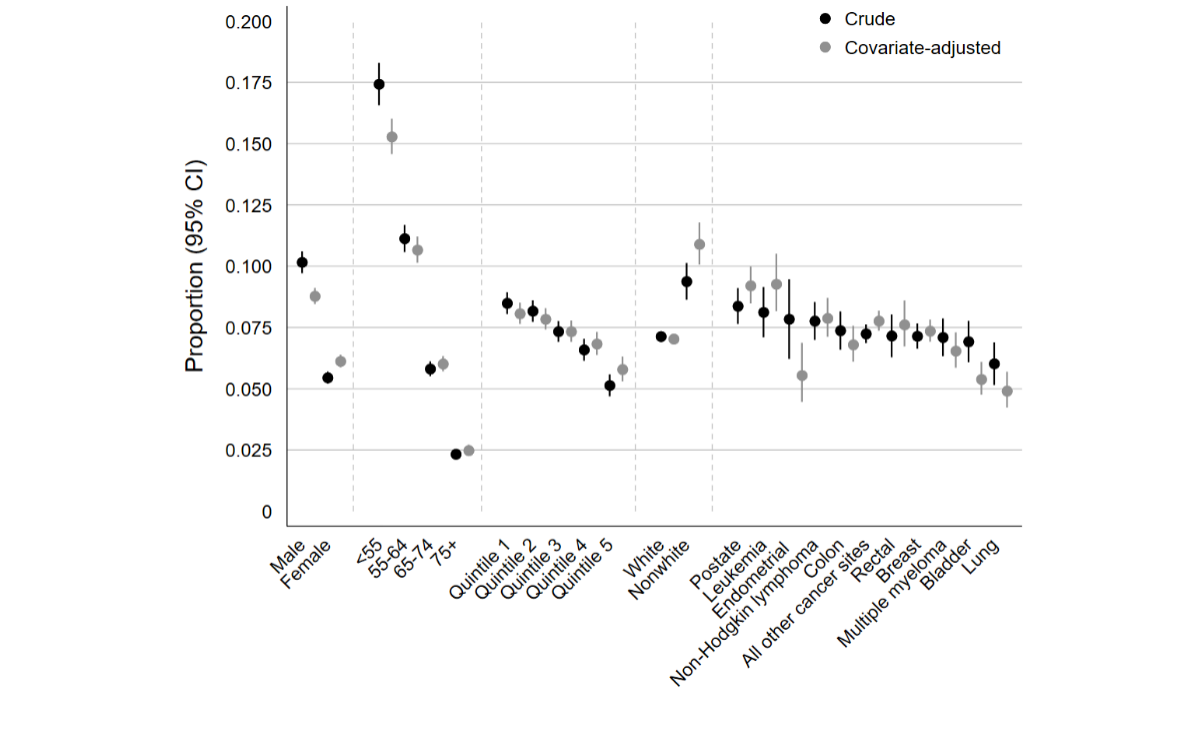
Predictors of online response: crude and covariate-adjusted proportions of online response.

Similar patterns of variation and related estimates to those obtained in the univariable analyses were also observed in the adjusted analysis, suggestive of an overall small degree of confounding between cancer site and sociodemographic variables (Table 1 and Figure 1).

### Associations Between Response Mode and Reported Experience

**Figure 2:**
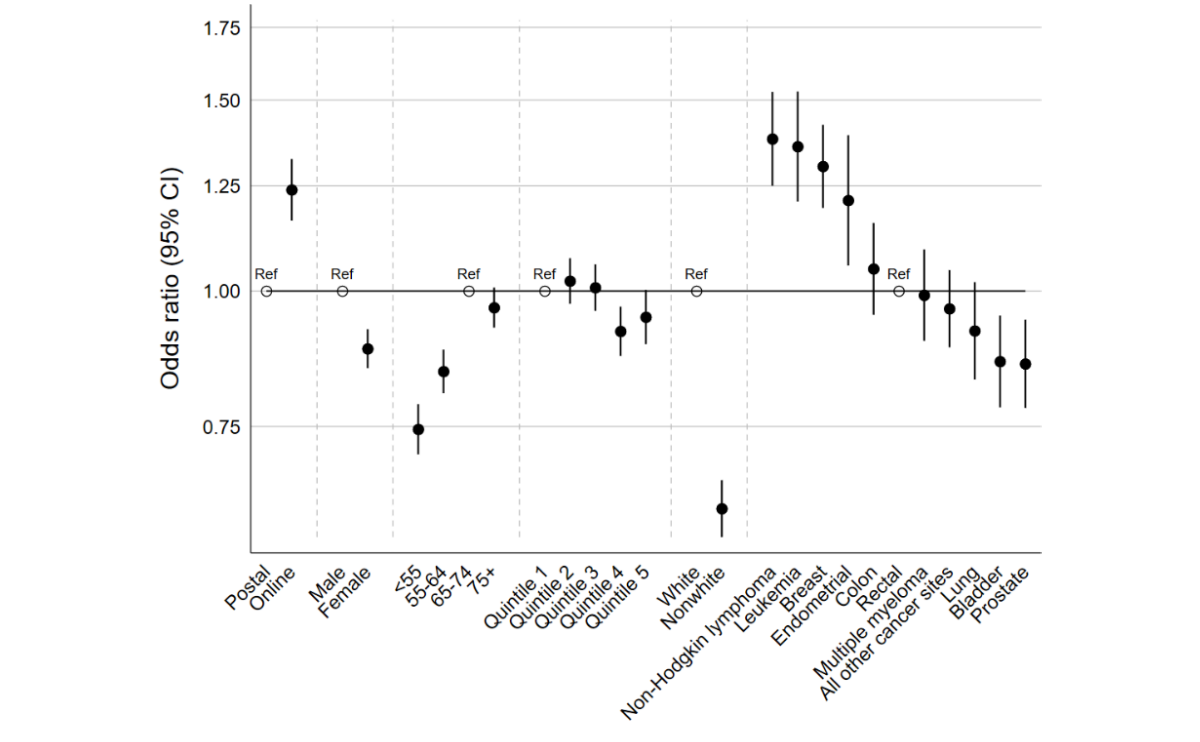
Associations between response mode and reporting a satisfied experience of cancer care (scores 9 or 10 to question 59): adjusted odds ratios of reporting a satisfied experience. Ref: reference category.

There were 60,921 patients in the analysis sample who answered the question about overall satisfaction with cancer care (#59). Of these, 22,030 (36.16%) patients gave a response in the “less than satisfied” category (defined as scores 0 to 8) and 38,891 (63.84%) patients responded in the “satisfied” category (defined as scores 9 or 10). Online responders were more likely to report a satisfied experience compared to those who responded by post (OR 1.16, 95% CI 1.09-1.24). Female, younger, more deprived, and nonwhite responders were more likely to report a less than satisfied experience (Table 2). There were also differences in overall satisfaction across cancer sites (joint *P* value <.001), with non-Hodgkin lymphoma being associated with the greatest likelihood and bladder cancer with the lowest likelihood of reporting a satisfied experience of care (Table 2 and Figure 2).

Adjusting for demographic characteristic, cancer site, and response mode variables led to some changes in the estimated associations for response mode, sex, and cancer site. In particular, adjustment accentuated the difference between online and postal responders (OR 1.24, 95% CI 1.16-1.32) (Table 2 and Figure 2).

**Figure 3:**
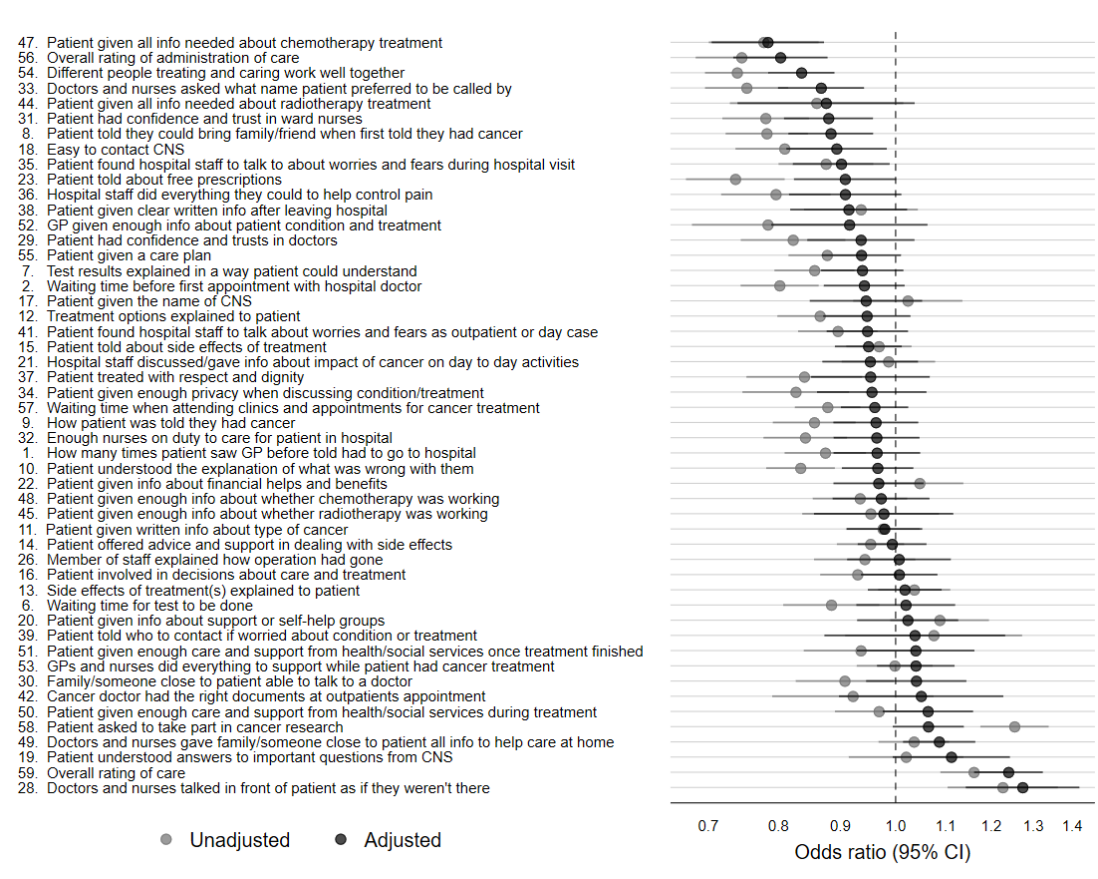
Associations between response mode and reported experience: adjusted odds ratios of reporting a satisfied/positive experience for online versus postal responders, by question. CNS: clinical nurse specialist; GP: general practitioner.

For each of the remaining 49 questions, the overall percentage of responders reporting a positive experience ranged from 28.89% (17,167/59,430) for question #58 (“patient asked to take part in cancer research”) to 95.63% (52,663/55,067) for question #42 (“cancer doctor had the right documents at outpatient appointment”) (Multimedia Appendix 3). Figure 3 presents the ORs of reporting a positive experience for responders who completed the questionnaire online compared with those who responded by post, both unadjusted and adjusted for patient demographic characteristic and cancer site variables. In general, the unadjusted associations tended to be more negative than the adjusted ones, possibly indicating a degree of confounding by the differences in the sociodemographic characteristics of online and postal responders, which became attenuated when age and other factors were taken into account.

Considering the adjusted analyses, there was evidence for an association between response mode and reported experience for 10 of 49 questions examined, although in opposite directions: for 8 questions, the online response mode was associated with a less than positive experience. These consisted of questions about “patient given all information needed about chemotherapy treatment” (#47), “overall rating of administration of care” (#56), “different people treating and caring work well together” (#54), “doctors and nurses asked what name patient preferred to be called by” (#33), “patient had confidence and trust in ward nurses” (#31), “patient told they could bring family/friend when first told they had cancer” (#8), “easy to contact clinical nurse specialist” (#18), and “patient found hospital staff to talk to about worries and fears during hospital visit” (#35). Conversely, for two questions online response mode was associated with a positive experience. Specifically, the questions about “doctors and nurses gave family/someone close to patient all information to help care at home” (#49) and “doctors and nurses talked in front of patient as if they were not there” (#28) (Figure 3).

**Table 1:** Predictors of online response: frequency and percentage of online response by patient characteristic and cancer site variables and related crude and adjusted odds ratios of online response (N=63,134).

Variable		Frequency and percentage of online response		Univariable logistic regression models		Multivariable logistic regression model	
		n (%)	Total, N	OR^a^ (95% CI)	*P* value^b^	aOR^c^ (95% CI)	*P* value^b^
**Sex**					<.001		<.001
	Male	2543 (8.78)	28,973	1		1	
	Female	2092 (6.12)	34,161	0.68 (0.64-0.72)		0.50 (0.46-0.54)	
**Age group (years)**					<.001		<.001
	<55	1457 (15.28)	9535	2.82 (2.61-3.05)		3.49 (3.21-3.80)	
	55-64	1377 (10.66)	12,913	1.87 (1.72-2.02)		2.05 (1.89-2.22)	
	65-74	1347 (6.01)	22,395	1		1	
	≥75	454 (2.48)	18,291	0.40 (0.36-0.44)		0.39 (0.35-0.43)	
**IMD score**					<.001		<.001
	Quintile 1 (least deprived)	1231 (8.06)	15,264	1		1	
	Quintile 2	1163 (7.84)	14,832	0.97 (0.89-1.05)		0.96 (0.88-1.04)	
	Quintile 3	1016 (7.33)	13,853	0.90 (0.83-0.98)		0.85 (0.78-0.93)	
	Quintile 4	749 (6.83)	10,963	0.84 (0.76-0.92)		0.75 (0.68-0.83)	
	Quintile 5 (most deprived)	476 (5.79)	8222	0.70 (0.63-0.78)		0.57 (0.51-0.64)	
**Ethnic group**					<.001		<.001
	White	4083 (7.03)	58,067	1		1	
	Nonwhite	552 (10.89)	5067	1.62 (1.47-1.78)		1.37 (1.24-1.51)	
**Cancer site^d^**					<.001		.01
	Prostate	527 (9.21)	5725	1.23 (1.05-1.44)		1.19 (1.01-1.41)	
	Leukemia	220 (9.27)	2373	1.24 (1.02-1.50)		1.15 (0.95-1.41)	
	Endometrial	78 (5.55)	1406	0.71 (0.55-0.93)		1.11 (0.84-1.46)	
	Non-Hodgkin lymphoma	350 (7.88)	4444	1.04 (0.87-1.23)		1.10 (0.92-1.31)	
	Colon	308 (6.80)	4530	0.89 (0.74-1.06)		1.03 (0.86-1.24)	
	Other cancers	1265 (7.76)	16,292	1.02 (0.88-1.18)		1.01 (0.87-1.18)	
	Rectal	235 (7.61)	3087	1		1	
	Breast	949 (7.35)	12,904	0.96 (0.83-1.12)		1.00 (0.85-1.18)	
	Multiple myeloma	298 (6.54)	4555	0.85 (0.71-1.02)		0.99 (0.83-1.19)	
	Bladder	239 (5.39)	4437	0.69 (0.57-0.83)		0.96 (0.80-1.17)	
	Lung	166 (4.91)	3381	0.63 (0.51-0.77)		0.83 (0.67-1.02)	
	Total	4635 (7.34)	63,134				

^a^Unadjusted odds ratios (ORs) of online response from a series of univariable logistic regression models, conditional on each patient characteristic and cancer site variable considered.

^b^*P* values from joint Wald tests.

^c^Adjusted odds ratios (aORs) of online response from a multivariable logistic regression model, conditional on all patient characteristic and cancer site variables considered.

^d^Responders with renal and thyroid cancers (grouped into the “other” category) and melanoma skin cancer excluded due to their ethnic group being suppressed or missing.

**Table 2:** Associations between response mode and reporting a satisfied experience of cancer care (scores 9 or 10 to question 59): frequency and percentage and related crude and adjusted odds ratios of reporting a satisfied experience (N=60,921).

Variable		Frequency and percentage of reporting a satisfied experience		Univariable logistic regression models		Multivariable logistic regression model	
		n (%)	Total, N	OR^a^ (95% CI)	*P* value^b^	aOR^c^ (95% CI)	*P* value^b^
**Response mode**					<.001		<.001
	Postal	35,809 (63.58)	56,318	1		1	
	Online	3082 (66.96)	4603	1.16 (1.09-1.24)		1.24 (1.16-1.32)	
**Sex**					.01		<.001
	Male	18,006 (64.36)	27,976	1		1	
	Female	20,885 (63.39)	32,945	0.96 (0.93-0.99)		0.89 (0.85-0.92)	
**Age group (years)**					<.001		<.001
	<55	5551 (59.73)	9294	0.77 (0.73-0.81)		0.75 (0.71-0.79)	
	55-64	7828 (62.18)	12,590	0.85 (0.81-0.89)		0.84 (0.81-0.88)	
	65-74	14,286 (65.88)	21,686	1		1	
	≥75	11,226 (64.70)	17,351	0.95 (0.91-0.99)		0.97 (0.93-1.01)	
**IMD score**					<.001		<.001
	Quintile 1 (least deprived)	9575 (64.82)	14,772	1		1	
	Quintile 2	9326 (65.10)	14,326	1.01 (0.97-1.06)		1.02 (0.97-1.07)	
	Quintile 3	8613 (64.41)	13,373	0.98 (0.94-1.03)		1.01 (0.96-1.06)	
	Quintile 4	6513 (61.65)	10,565	0.87 (0.83-0.92)		0.92 (0.87-0.97)	
	Quintile 5 (most deprived)	4864 (61.69)	7885	0.87 (0.83-0.93)		0.95 (0.89-1.00)	
**Ethnic group**					<.001		<.001
	White	36,343 (64.80)	56,088	1		1	
	Nonwhite	2548 (52.72)	4833	0.61 (0.57-0.64)		0.63 (0.59-0.67)	
**Cancer site^d^**					<.001		<.001
	Non-Hodgkin lymphoma	3025 (70.25)	4306	1.36 (1.23-1.50)		1.38 (1.25-1.53)	
	Leukemia	1604 (69.86)	2296	1.34 (1.19-1.50)		1.36 (1.21-1.53)	
	Breast	8257 (65.95)	12,521	1.12 (1.03-1.21)		1.30 (1.19-1.42)	
	Endometrial	880 (65.67)	1340	1.10 (0.96-1.26)		1.21 (1.06-1.39)	
	Colon	2800 (64.35)	4351	1.04 (0.94-1.15)		1.05 (0.95-1.16)	
	Rectal	1898 (63.44)	2992	1		1	
	Multiple myeloma	2788 (63.31)	4404	0.99 (0.90-1.10)		0.99 (0.90-1.09)	
	Other	9634 (61.39)	15,692	0.92 (0.85-0.99)		0.96 (0.89-1.05)	
	Lung	2002 (61.45)	3258	0.92 (0.83-1.02)		0.92 (0.83-1.02)	
	Bladder	2600 (61.19)	4249	0.91 (0.83-1.00)		0.86 (0.78-0.95)	
	Prostate	3403 (61.74)	5512	0.93 (0.85-1.02)		0.86 (0.78-0.94)	
	Total	38,891 (63.84)	60,921				

^a^Unadjusted odds ratios (ORs) of reporting a satisfied experience from a series of univariable logistic regression models, conditional on each of the response mode, patient characteristic and cancer site variables considered.

^b^*P* values from joint Wald tests.

^c^Adjusted odds ratios (aORs) of reporting a satisfied experience from a multivariable logistic regression model, conditional on response mode and all patient characteristic and cancer site variables considered.

^d^Responders with renal and thyroid cancers (grouped into the “other” category) and melanoma skin cancer excluded due to their ethnic group being suppressed or missing.

Multimedia Appendix 4 presents the variation in observed *P* values for the association between online response mode and reported experience for all 50 questions (49 aspect-specific questions plus the question addressing overall satisfaction) after adjustment for patient characteristic and cancer site variables. The observed variation is compared with what might be expected under the null hypothesis of no association (straight line). If there were no true associations, then two to three questions would be expected to have a *P* value less than .05 (dashed line) by chance alone, and the observed distribution would follow the straight line. The distribution of *P* values across these questions suggests that it is unlikely that online response mode was unrelated to reported experience.

### Supplementary Analyses

When considering the association between response mode and overall satisfaction with cancer care, defining an alternative cut-off point for overall satisfaction (scores 8 to 10) yielded broadly comparable results to that using the original cut-off point (scores 9 or 10) (Multimedia Appendix 5). For this item, there was also strong evidence of an interaction between response mode and sex, as well as between response mode and ethnic group (*P* value for each interaction <.001). In contrast, there was no evidence for an interaction between response mode and any of the age group, social deprivation, or cancer site variables. Therefore, although for both response modes women were less likely than men to report a satisfied experience of care, this difference by sex was stronger for female online responders (OR 0.76, 95% CI 0.67-0.86 versus men) compared with female postal responders (OR 0.89, 95% CI 0.86-0.93 versus men). Furthermore, although nonwhite postal responders were less likely to report a satisfied experience of their care (OR 0.60, 95% CI 0.56-0.64 versus white ethnic group), there was no evidence for such a difference among nonwhite online responders (OR 0.94, 95% CI 0.78-1.14 versus white ethnic group) (Multimedia Appendix 6).

## Discussion

### Summary of Findings

Using data from a major English experience survey of patients recently treated with cancer, we found that a notable minority (approximately 1 in 14) of all participants responded to the survey online, with male, younger, least deprived, and nonwhite patients being more likely to do so. However, when examining associations between response mode and aspects of cancer patient experience, we observed statistically nonsignificant associations, with point estimates generally indicative of less frequent positive ratings for online responders. There were some exceptions; we found three questions with evidence that online responders were more likely to respond positively, particularly the question on overall satisfaction with care.

### Strengths and Limitations

This study was based on the analysis of a large nationwide sample of responders, which allowed us to examine potential interactions, beyond main effect analyses, and we were able to adequately adjust for a range of patient characteristics (namely age group, sex, deprivation, ethnic group, and cancer site) of known relevance to patient experience surveys [15-17]. An important limitation is that we were unable to directly examine how reported experience related to actual care experience. As such, we could not establish whether the online response mode affected how people reported their experience of care, or whether patients who would have provided lower or higher ratings of care (regardless of response mode) were more likely to respond online. The challenge of drawing appropriate inferences in respect of this research question is further complicated by the fact that the associations between response mode and patient experience that we examined were heterogeneous in their presence, direction, and size. Another limitation of our analysis is that we had no means of examining potential differences by response mode in usability (eg, the time taken to complete and post or submit the survey questionnaire).

To aid interpretation of findings, we conducted a post hoc comparison of how each question appeared in the postal and online questionnaires. For this, we used the online demo questionnaire of the 2017 survey provided by the survey provider (Quality Health), which was identical in format to that of the 2015 survey, and the published postal questionnaire of the 2015 survey. Allowing for the difference in the medium, we found that the presentation of items was identical between the postal and online questionnaires, except for the question regarding overall satisfaction, which was presented slightly differently. In particular, the anchoring text for this question on the postal version covered scores 8 to 10 for “very good”, whereas it only covered 10 on the online version, with similar differences at the opposite end of the scale (Multimedia Appendix 7). This discrepancy in the appearance of the question might contribute to explaining the difference in overall rating of satisfaction between postal and online responders. However, we urge caution in this interpretation because this item is not particularly unusual in its association with online response mode, considering the distribution of associations observed for the other items (Figure 3).

### Findings in Relation to Other Evidence and Implications

We were not aware of any relevant literature on the use of an online response option specifically in cancer patient experience surveys. However, a previous German study that examined health behaviors using data from a population-based longitudinal panel reported an overall equivalence in responses obtained from the Web-only response mode arm compared with a mixed mode (paper or Web) arm [18].

Although not the principal focus of our inquiry, we confirmed a previously identified variation in the association between sociodemographic characteristics and cancer site with rated satisfaction or experience of cancer care [15,19]. The findings of a higher probability of online response by male, younger, and less deprived patients might be expected, but nonwhite patients were also more likely to respond online. This observation contrasts with the patterns of variation by ethnic group in use of the internet among members of the general population [20]. Further research to help understand this ethnic variation is needed.

In survey research, it is generally important to consider whether response mode ought to be adjusted for when examining sociodemographic inequalities, trends over time, or when considering organizational comparisons [21]. The answer to this research question depends on the direction of causality between response mode and reported patient experience. We advocate the need for high-definition experimental studies in small, yet adequately powered, subsamples of responders who will be otherwise matched for all their characteristics except response mode. For example, Elliott et al [10] conducted a randomized controlled trial to examine the influence of survey response mode on experience ratings in the context of the Consumer Assessments of Healthcare Providers and Systems Hospital Survey. The results suggested the need for adjusting for survey response mode in the calculation of hospital scores. Unlike other forms of randomized controlled trials that are associated with substantial practical and ethical barriers, such trials are relatively easy to conduct in the context of survey research.

### Conclusions

We described the sociodemographic and cancer site predictors of the online response option in a major national survey in England, and examined potential associations of response mode with rated satisfaction and experience of cancer care. The findings highlighted that online and postal responders differed in their patient characteristics, with less evidence for variation between online and postal responders in terms of experience ratings. Whether the association between response mode and satisfaction with care is causal in the context of cancer patient experience surveys needs to be examined experimentally.

## Appendix

Multimedia Appendix 1 Summary of sociodemographic variables among patients with suppressed ethnic group.

Multimedia Appendix 2 Analysis sample derivation.

Multimedia Appendix 3 Associations between response mode and reported experience: question-specific sample size, percentage of a satisfied/positive experience, and odds ratio of reporting a satisfied/positive experience for online versus postal responders, adjusted for patient characteristic and cancer site variables, by question. CNS: clinical nurse specialist; GP: general practitioner.

Multimedia Appendix 4 Associations between response mode and reported experience: variation in observed P values for the association between online response mode and reported experience for 50 examined questions.

Multimedia Appendix 5 Supplementary analyses: adjusted odds ratios of reporting a satisfied experience of cancer care (scores 8 to 10 to question 59). Ref: reference category.

Multimedia Appendix 6 Supplementary analyses: adjusted odds ratios of reporting a satisfied experience of cancer care (scores 9 or 10 to question 59) with the inclusion of pairwise interactions of response mode with sex and ethnic group (N=60,921).

Multimedia Appendix 7 Associations between response mode and reported experience: comparison of the presentation of question 59 on overall satisfaction with cancer care in the postal and online questionnaires.

## Abbreviations

CI: confidence interval
CPES: Cancer Patient Experience Survey
IMD: Index of Multiple Deprivation
NHS: National Health Service
OR: odds ratio
