# Supplementary material for: Predictors of Postal or Online Response Mode and Associations With Patient Experience and Satisfaction in the English Cancer Patient Experience Survey
Source: J Med Internet Res. 2019 May 2;21(5):e11855. doi: 10.2196/11855 (PMC6521193; doi:10.2196/11855)
Supplement: Multimedia Appendix 1 [file jmir_v21i5e11855_app1.pdf]

Variable	n	%
Age group		
<55	647	21.12
55-64	597	19.48
65-74	924	30.16
≥75	896	29.24
Sex		
Male	1591	51.93
Female	1473	48.07
IMD score		
Quintile 1	803	26.21
Quintile 2	728	23.76
Quintile 3	646	21.08
Quintile 4	530	17.30
Quintile 5	332	10.83
Missing	25	0.82
Cancer site		
Melanoma	1633	53.30
Renal[a]	938	30.61
Thyroid[a]	493	16.09
Total	3064	100.00

[a] Renal and thyroid cancers were grouped into the "other" category.
