# Supplementary figures and images for: Predictors of Postal or Online Response Mode and Associations With Patient Experience and Satisfaction in the English Cancer Patient Experience Survey
Source: J Med Internet Res. 2019 May 2;21(5):e11855. doi: 10.2196/11855 (PMC6521193; doi:10.2196/11855)

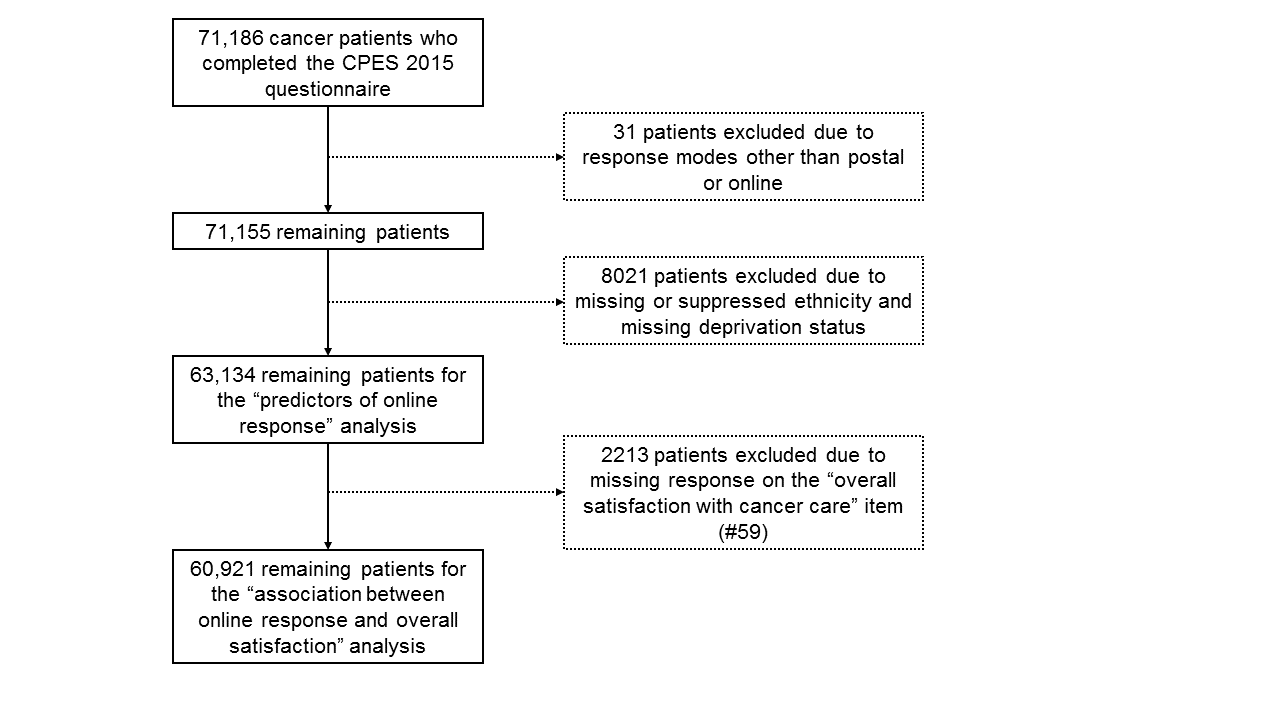

Supplement: Multimedia Appendix 2 [file jmir_v21i5e11855_app2.png]

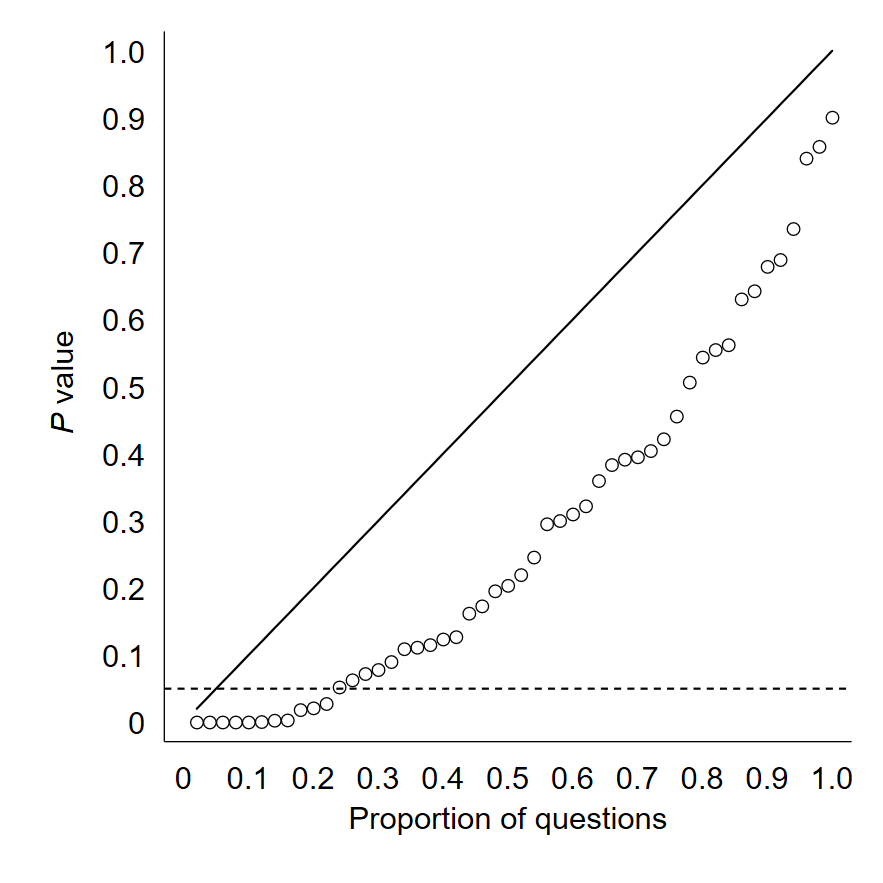

Supplement: Multimedia Appendix 4 [file jmir_v21i5e11855_app4.png]

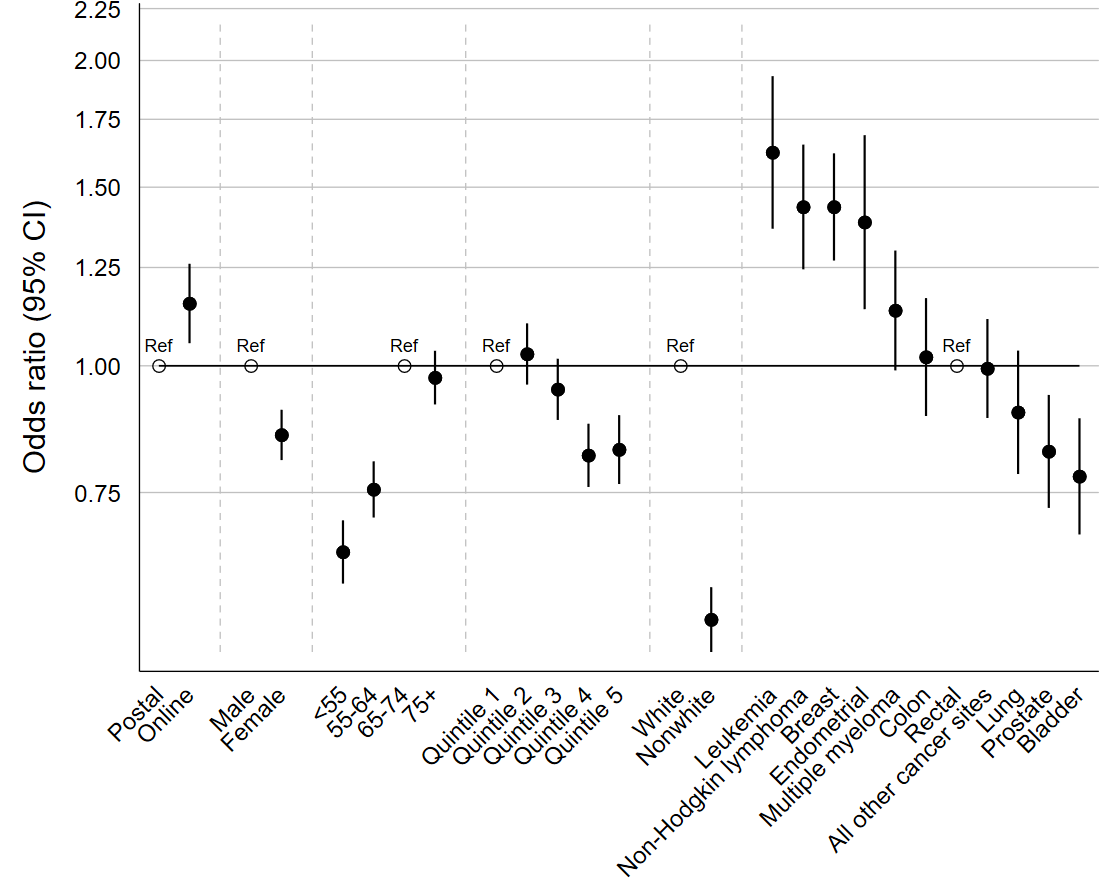

Supplement: Multimedia Appendix 5 [file jmir_v21i5e11855_app5.png]
