# Supplementary material for: Predictors of Postal or Online Response Mode and Associations With Patient Experience and Satisfaction in the English Cancer Patient Experience Survey
Source: J Med Internet Res. 2019 May 2;21(5):e11855. doi: 10.2196/11855 (PMC6521193; doi:10.2196/11855)
Supplement: Multimedia Appendix 3 [file jmir_v21i5e11855_app3.pdf]

Question and synopsis	N	Overall % of reported satisfied/positive experience	Odds ratio[a]	95% confidence interval	*P* value[b]
Q47 Patient given all info needed about chemotherapy treatment	33,454	84.41	0.78	0.70 - 0.87	**<.001**
Q56 Overall rating of administration of care	61,879	89.01	0.80	0.73 - 0.88	**<.001**
Q54 Different people treating and caring work well together	60,147	60.45	0.84	0.78 - 0.89	**<.001**
Q33 Doctors and nurses asked what name patient preferred to be called by	38,788	66.52	0.87	0.80 - 0.94	**.001**
Q44 Patient given all info needed about radiotherapy treatment	16,558	86.02	0.88	0.74 - 1.04	.124
Q31 Patient had confidence and trust in ward nurses	39,117	72.21	0.88	0.81 - 0.96	**.003**
Q8 Patient told they could bring family/friend when first told they had cancer	52,096	79.08	0.88	0.82 - 0.96	**.003**
Q18 Easy to contact CNS	48,330	86.58	0.89	0.81 - 0.98	**.021**
Q35 Patient found hospital staff to talk to about worries and fears during hospital visit	28,833	52.08	0.90	0.82 - 0.99	**.028**
Q23 Patient told about free prescriptions	29,538	80.70	0.91	0.82 - 1.00	.052
Q36 Hospital staff did everything they could to help control pain	34,333	83.96	0.91	0.82 - 1.01	.078
Q38 Patient given clear written info after leaving hospital	36,697	84.55	0.91	0.82 - 1.02	.115
Q52 GP given enough info about patient condition and treatment	52,266	95.51	0.92	0.79 - 1.06	.246
Q29 Patient had confidence and trusts in doctors	39,106	84.06	0.94	0.84 - 1.04	.204
Q55 Patient given a care plan	48,025	32.94	0.94	0.87 - 1.01	.090
Q7 Test results explained in a way patient could understand	53,637	78.80	0.94	0.87 - 1.01	.111
Q2 Waiting time before first appointment with hospital doctor	61,529	82.25	0.94	0.87 - 1.02	.127
Q17 Patient given the name of CNS	60,053	90.29	0.94	0.85 - 1.05	.300
Q12 Treatment options explained to patient	54,397	82.61	0.95	0.87 - 1.03	.195
Q41 Patient found hospital staff to talk about worries and fears as outpatient or day case	46,824	70.18	0.95	0.88 - 1.02	.173
Q15 Patient told about side effects of treatment	56,469	54.20	0.95	0.89 - 1.01	.109
Q21 Hospital staff discussed/gave info about impact of cancer on day to day activities	41,729	81.07	0.95	0.87 - 1.04	.295
Q37 Patient treated with respect and dignity	39,086	87.51	0.95	0.85 - 1.07	.404
Q34 Patient given enough privacy when discussing condition/treatment	39,051	84.75	0.96	0.86 - 1.06	.391
Q57 Waiting time when attending clinics and appointments for cancer treatment	61,369	65.73	0.96	0.90 - 1.02	.220
Q9 How patient was told they had cancer	62,079	84.15	0.96	0.89 - 1.04	.360
Q32 Enough nurses on duty to care for patient in hospital	38,943	65.84	0.96	0.89 - 1.05	.383
Q1 How many times patient saw GP before told had to go to hospital	46,107	75.46	0.96	0.89 - 1.05	.395
Q10 Patient understood the explanation of what was wrong with them	62,283	72.68	0.97	0.90 - 1.03	.322
Q22 Patient given info about financial helps and benefits	33,276	55.32	0.97	0.89 - 1.05	.456

Q48 Patient given enough info about whether chemotherapy was working	30,433	67.82	0.97	0.89 - 1.07	.555
Q45 Patient given enough info about whether radiotherapy was working	14,080	59.34	0.98	0.86 - 1.12	.735
Q11 Patient given written info about type of cancer	54,868	71.61	0.98	0.91 - 1.05	.562
Q14 Patient offered advice and support in dealing with side effects	59,744	66.27	0.99	0.93 - 1.06	.840
Q26 Member of staff explained how operation had gone	32,975	77.43	1.01	0.91 - 1.11	.901
Q16 Patient involved in decisions about care and treatment	60,325	77.59	1.01	0.94 - 1.08	.857
Q13 Side effects of treatment(s) explained to patient	60,115	72.82	1.02	0.95 - 1.09	.630
Q6 Waiting time for test to be done	53,505	86.71	1.02	0.93 - 1.12	.689
Q20 Patient given info about support or self-help groups	46,879	83.25	1.02	0.93 - 1.13	.642
Q39 Patient told who to contact if worried about condition or treatment	37,664	93.91	1.04	0.87 - 1.23	.679
Q51 Patient given enough care and support from health/social services once treatment finished	19,365	44.43	1.04	0.93 - 1.16	.506
Q53 GPs and nurses did everything to support while patient had cancer treatment	43,561	62.41	1.04	0.97 - 1.12	.310
Q30 Family/someone close to patient able to talk to a doctor	32,567	71.91	1.04	0.95 - 1.14	.422
Q42 Cancer doctor had the right documents at outpatients appointment	55,067	95.63	1.05	0.90 - 1.23	.543
Q50 Patient given enough care and support from health/social services during treatment	33,215	53.68	1.06	0.98 - 1.16	.162
Q58 Patient asked to take part in cancer research	59,430	28.89	1.06	0.99 - 1.14	.072
Q49 Doctors and nurses gave family/someone close to patient all info to help care at home	50,433	57.43	1.09	1.01 - 1.16	**.018**
Q19 Patient understood answers to important questions from CNS	46,283	88.61	1.11	0.99 - 1.24	.063
Q59 Overall rating of care	60,921	63.84	1.24	1.16 - 1.32	**<.001**
Q28 Doctors and nurses talked in front of patient as if they weren't there	39,018	81.52	1.27	1.14 - 1.42	**<.001**

[a] Odds ratios of reporting a satisfied/positive experience for online versus postal responders from multivariate logistic regression models, adjusted for age group, sex, ethnic group, social deprivation, and cancer site variables.

[b] *P* value from Wald tests, highlighted at 5% level.
