# Supplementary material for: Predictors of Postal or Online Response Mode and Associations With Patient Experience and Satisfaction in the English Cancer Patient Experience Survey
Source: J Med Internet Res. 2019 May 2;21(5):e11855. doi: 10.2196/11855 (PMC6521193; doi:10.2196/11855)
Supplement: Multimedia Appendix 6 [file jmir_v21i5e11855_app6.pdf]

Variable	Odds ratio[a]	95% confidence interval	*P* value[b]
Response mode			
Postal	1		<.001
Online	1.27	1.15 - 1.39	
Interaction			
Online # female	0.85	0.75 - 0.97	<.001
Online # nonwhite	1.56	1.28 - 1.91	<.001
Sex			
Male	1		<.001
Female	0.89	0.86 - 0.93	
Age group			
<55	0.75	0.71 - 0.79	<.001
55-64	0.84	0.81 - 0.88	
65-74	1		
75+	0.97	0.93 - 1.01	
IMD score			
Quintile 1	1		<.001
Quintile 2	1.02	0.97 - 1.07	
Quintile 3	1.01	0.96 - 1.06	
Quintile 4	0.92	0.87 - 0.97	
Quintile 5	0.95	0.89 - 1.00	
Ethnic group			
White	1		<.001
Nonwhite	0.60	0.56 - 0.64	
Cancer site[c]			
Non-Hodgkin lymphoma	1.38	1.25 - 1.52	<.001
Leukemia	1.35	1.20 - 1.52	
Breast	1.30	1.19 - 1.42	
Endometrial	1.21	1.06 - 1.39	
Colon	1.05	0.95 - 1.15	
Rectal	1		
Multiple myeloma	0.99	0.90 - 1.09	
Other	0.96	0.89 - 1.04	
Lung	0.92	0.83 - 1.02	
Bladder	0.86	0.78 - 0.95	
Prostate	0.86	0.78 - 0.94	

[a] Adjusted odds ratios of reporting a satisfied experience from a multivariable logistic regression model, conditional on response mode, patient characteristic and cancer site variables, as well as pairwise interaction terms for response mode with sex and ethnic group.

[b] *P* values from joint Wald tests.

[c] Responders with renal and thyroid cancers (grouped into the “other” category) and melanoma skin cancer excluded due to their ethnic group being suppressed or missing.
