# Supplementary material for: Predictors of Postal or Online Response Mode and Associations With Patient Experience and Satisfaction in the English Cancer Patient Experience Survey
Source: J Med Internet Res. 2019 May 2;21(5):e11855. doi: 10.2196/11855 (PMC6521193; doi:10.2196/11855)
Supplement: Multimedia Appendix 7 [file jmir_v21i5e11855_app7.pdf]

a. Postal questionnaire

59. Overall, how would you rate your care?
(**Please circle a number**)

Very poor Very good

0 1 2 3 4 5 6 7 8 9 10

b. Online questionnaire (taken from the demo version of the 2017 online questionnaire with identical format to the 2015 questionnaire)

59. Overall, how would you rate your care?

Very poor 0	1	2	3	4	5	6	7	8	9	Very good 10
☐	☐	☐	☐	☐	☐	☐	☐	☐	☐	☐
